# Epidemiological features of cutaneous leishmaniasis and distribution of sand flies in an endemic area in southeast of Iran

**DOI:** 10.1016/j.parepi.2021.e00220

**Published:** 2021-07-28

**Authors:** Alireza Sanei-Dehkordi, Moussa Soleimani-Ahmadi, Mehdi Zare, Hadi Mirzaei

**Affiliations:** a Department of Medical Entomology and Vector Control, Faculty of Health, Hormozgan University of Medical Sciences, Bandar Abbas, P.O. Box: 79145–3838, Iran; b Social Determinants in Health Promotion Research Center, Hormozgan University of Medical Sciences, Bandar Abbas, Iran; c Department of Occupational Health Engineering, Faculty of Health, Hormozgan University of Medical Sciences, Bandar Abbas, Iran; d Hajiabad Health Center, Hormozgan University of Medical Sciences, Hajiabad, Iran

**Keywords:** Cutaneous leishmaniasis, Epidemiology, Sand fly, Hajiabad, Iran

## Abstract

**Introduction:**

Cutaneous leishmaniasis (CL) is a widespread tropical infectious disease in the world. It is one of the most important health problem in Iran which is endemic in different parts of country. This study was conducted to determine epidemiological features of CL and distribution of sand flies in Hajiabad County, one of the important CL foci in southeast of Iran.

**Methods:**

This descriptive cross-sectional study was conducted from March 2019 to March 2020.

All of the suspected CL cases with skin lesions who referred to Hajiabad County health centers and all actively detected patients were clinically and parasitologically examined for CL. Demographic and clinical data of all patients were recorded. Moreover, in this study, sand flies were collected monthly from four typical plain and foothill villages during their active season (May–November) using sticky trap. Sand flies were mounted as permanent microscopic slides, using Puri's medium, and identified by taxonomic keys. Data were analyzed using SPSS.21 software and descriptive statistics.

**Results and discussion:**

A total of 70 confirmed cases of CL were recorded; the incidence rate of the disease was 101 per 100,000 people. The most infected age group was 0–10 years, with a rate of 64.3%. Males were infected more than females and the majority of cases (85.7%) were recorded from rural areas. Most of the cases had 1 lesion (51.4%) and the most lesions (55.8%) were in upper extremities. During the study period, 832 sand fly specimens comprised of ten species of *Sergentomyia* and seven of *Phlebotomus* were collected and identified. The most prevalent species was *P. papatasi* (47.12%), followed by *P. alexandri* (8.41%) and *P. salehi* (6.25%). Among the collected *Phlebotomus* species, *P. papatasi* and *P. sergenti* are known as the primary vectors of CL and *P. alexandri*, *P. salehi*, and *P. caucasicus*, play the main role as the secondary vectors of CL in Iran.

**Conclusion:**

This study has revealed that CL is endemic in Hajiabad County and there are five CL vectors that are distributed in this County and some of them are more prevalent in plain areas. These findings can be used as a basis for implementation of interventions toward vector control, which may help in suppression of vector density, and consequently, control of CL in the study area.

## 1 Introduction

Cutaneous leishmaniasis (CL) is one of the important vector-borne diseases which is caused by several species of obligate protozoan parasites from the genus *Leishmania* and transmitted by phlebotomine sand flies (Akhoundi et al., 2016). It is endemic in more than 70 countries and approximately 12 million humans are infected, and about 600, 000 to 1 million new cases of CL occur each year worldwide (WHO, 2020). According to the WHO report, about 85% of CL cases were reported from Iran, Afghanistan, Pakistan, Iraq, Syria, Tunisia, Peru, Bolivia, Algeria, Colombia, and Brazil (WHO, 2020).

CL is one of the serious health problems in Iran, which is widely distributed and has been reported from 25 of 31 Provinces of the country (Ghatee et al., 2020). The annual burden of CL in Iran is about 22,000 cases and approximately 80% of these cases are zoonotic cutaneous leishmaniasis (ZCL) (Karimi et al., 2014). The prevalence of CL in different provinces of Iran has been reported from 1.8% to 37.9% (Khazaei et al., 2015). There are two epidemiological forms of CL in Iran including ZCL and anthroponotic cutaneous leishmaniasis (ACL). ZCL is caused by *L.major* and is endemic in 15 out of 31 provinces of Iran (Shamsian et al., 2020). The main reservoirs of ZCL are desert rodents, including *Tatera indica*, *Meriones libycus*, *M. persicus*, *M. hurrianae*, *Rhombomys opimus*, and *Nesokia indica* (Yaghoobi-Ershadi, 2012; Gholamrezaei et al., 2016). According to the results of studies conducted in Iran, ZCL has two transmission cycles including wild (zoonotic) and domestic cycle. In the wild cycle transmission, the sand flies of the *Phlebotomus caucasicus* group, including *P. mongolensis*, *P. caucasicus*, and *P. andrejievi,* transmit the *Leishmania* parasite among wild rodent population (wild rodent-sand fly-wild rodent). But, in the domestic cycle, the vector is *P. papatasi,* which transmits the parasite (*Leishmania major*) from an infected wild rodent/human to a susceptible wild rodent/human (Karimi et al., 2014; Yaghoobi-Ershadi, 2012; Yaghoobi-Ershadi and Javadian, 1996; Parvizi et al., 2012).

ZCL is mostly endemic in rural arid areas and according to the distribution of reservoir hosts, the disease has four main foci in Iran which are located in centre and northeast, west and southwest, southeast, and some villages of Fars Province in south of the Iran (Yaghoobi-Ershadi, 2012; Sofizadeh et al., 2018a).

In recent decades a significant increase in the number of leishmaniasis cases has been reported worldwide (Alvar et al., 2012). The increase may be due to the result of environmental and behavioural changes such as deforestation, large migrations from rural to urban areas, unplanned urbanization, new irrigation projects, and building of dams which increase exposure of humans to sand fly vectors (Oryan and Akbari, 2016; Desjeux, 2001). Therefore, it is of importance to identify new ZCL foci to implement appropriate control measures to prevent the spread of the disease to new areas in the future.

In the Hormozgan province, the CL is more prevalent and the incidence rate of disease in the province has been 22/100,000 during the last 3 decades, although the incidence has decreased to 6.39/100,000 in the recent years (Hanafi-Bojd et al., 2018).

Hajiabad County is one of the ZCL foci in Hormozgan Province and the circulation of L. *major* in humans, gerbils, and *P. papatasi* has been documented in the County (Hanafi-Bojd et al., 2018; HanafiBojd et al., 2006). Continuous monitoring of epidemiological aspects of CL is necessary for proper decisions toward effective interventions in the area. The current study was conducted to determine epidemiological features of CL and distribution of sand flies in Hajiabad County, one of the most important CL foci in southeast of Iran.

## 2 Material and methods

### 2.1 Study areas

The study was conducted in Hajiabad, a County in the Hormozgan province, southeast of Iran. This County has an area of 9459 km^2^ and is located between longitudes 55°14′-57°01′ E and latitudes 27°24′-28°53′ N, with 69,265 populations according to 2016 census from which 50.8% were males and 49.2% females. The area is mountainous with low level of precipitation. It has dry summers and moderate winters. The average annual rainfall in this county, is 208 mm and the averages of minimum and maximum relative humidity are 15% in August and 23% in November, respectively. In this region, the climate is tropical and the mean annual temperature is 22.9 °C and ranging from 12 to 35.1 °C. Agriculture, animal husbandry are the main occupations in this area. Major products of this County are vegetables, citrus fruits and dates.

### 2.2 Study design and data collection

The current descriptive cross-sectional study was conducted from March 2019 to March 2020 in Hajiabad County. In the study, demographic and clinical data of all CL cases, who referred to local health centers for treatment and follow up were recorded. In health centers all of suspicious CL patients were examined and parasitological verifications were applied to diagnose the CL. In this regard, simple direct smears were taken from the ulcers, in order to identify amastigote forms of the parasite. Then, the suspected samples were placed on a slide and stained by Giemsa stain. In the final stage, the slides were tested using a light microscope. After the examination, all of microscopic approved cases were interviewed using a structured and pre-tested questionnaire. The questionnaires were completed by trained interviewers and supervised by the chief investigator (M.S.-A). The questionnaire contained demographic characteristics such as gender, place of residence (rural, urban) and clinical information including size, number, and location of the lesions.

### 2.3 Sand fly collection and species identification

**Fig. 1:**
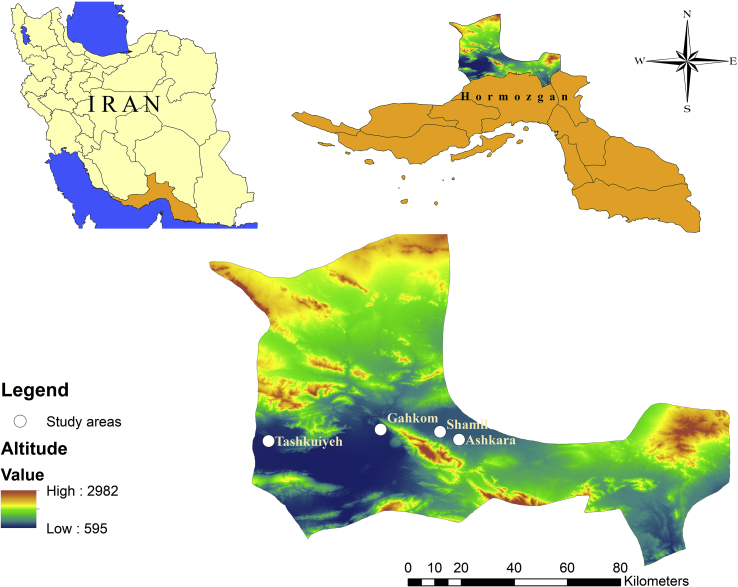
Map showing the provinces of Iran, highlighting the location of Hormozgan Province and sand flies sampling locations in Hajiabad County, southeast of Iran.

In this study, based on the reported CL cases during the past years, suitability of the locations for sand flies collection, and human population densities, four villages in different topographical areas in Hajiabad County were randomly selected. The selected villages included: Tashkuiyeh (28°14′N, 55°44′E, 682 m) and Ashkara (28°14′N, 56°09′E, 85 m) in plain areas and Shamil (28°14′N, 56°09′E, 857 m) and Gahkom (28°17′N, 55°82′E, 693 m) in foothill areas (Fig. 1).

Sand flies were collected in 2019 during their active season (May–November) using sticky trap (castor oil coated white papers, 20 × 32 cm) from indoors (bedrooms, warehouses, toilets, bathrooms, etc.) and outdoors (rodent burrows) in the selected villages of Hajiabad County. For each sampling, 60 sticky traps (30 outdoors, 30 indoors) were laid before sunset and collected next early morning (before the following sunrise). Specimens were removed from the traps, rinsed in the acetone, and then conserved in 70% ethanol. Sand flies were mounted as permanent microscopic slides, using Puri's medium, and identified by morphological keys (Theodor and Mesghali, 1964; Seyedi and Nadim, 1992).

### 2.4 Statistical analysis

To analyze the data, SPSS ver.21 software was used. Descriptive statistics were used to show the percentages, averages, and relative frequencies.

The *t*-test and chi-square test were used to analyze the data. The results were considered significant at 5% level (*p*-value <0.05).

## 3 Results and discussion

### 3.1 Epidemiological features of cutaneous leishmaniasis

During this study, a total of 70 laboratory-confirmed cases of CL were recorded in Hajiabad County. Accordingly, the annual incidence rate of CL in this County was 101per 100,000 population, which is higher than the average incidence rate of the disease in Iran which is between 20 and 40 cases per 100,000 population (Shirzadi, 2012). In contrast, the incidence rate of CL in Hajiabad County is lower compared to the endemic Provinces such as Yazd, Bushehr, Fars, Ilam, Khuzestan, Khorasan Razavi, and Isfahan, where the average of CL incidence has been reported to be 166 cases per 100,000 population [19–21].

**Fig. 2:**
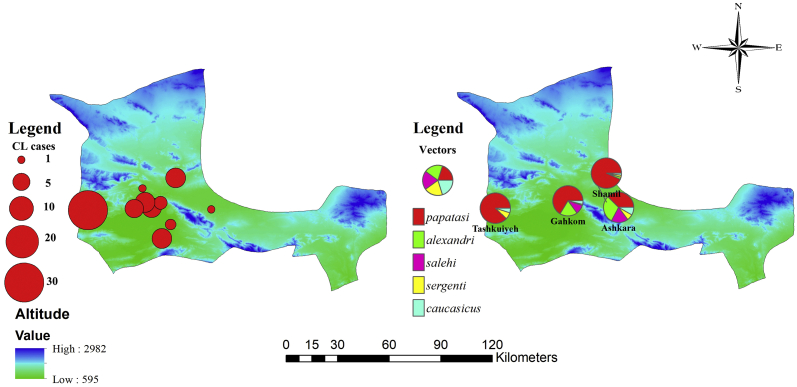
Spatial distribution of cutaneous leishmaniasis (a) and relative abundance of vector sand flies (b) in Hajiabad County, southeast of Iran, 2019.

Spatial distribution of CL patients in the Hajiabad County showed that the highest morbidity was in the south-western regions (Fig. 2). Overall, the disease was recorded in 10 villages and Hajiabad city.

Men were infected more than women, with 45.7% of cases occurring in women over the studied period. This finding is in accordance with other epidemiologic studies in Iran which revealed higher incidence rate of CL in men (Rostami et al., 2013; Mohammadi and Soltani, 2019; Nilforoushzadeh et al., 2014; Rezaee et al., 2020; Akhlagh et al., 2019). Similar findings have been reported from other endemic countries such as Saudi Arabia, Pakistan, Iraq, Syria, Jordan, Yemen, Libya, Algeria, Bolivia, and Brazil, where males had higher CL infection than females (Abuzaid et al., 2017; Khan et al., 2016; AlSamarai and AlObaidi, 2009; Youssef et al., 2019; Salam et al., 2014; Alkulaibi et al., 2019; Khezzani and Bouchemal, 2017; Eid et al., 2018; Araujo et al., 2016; Krieger, 2003). The difference in risk of CL infection between women and men is related to differences in gender roles and not to biological characteristics associated with sex (Khademvatan et al., 2017). In this regard, social activities, type of clothing, and the presence of men outside the home, increase their contact with infective sand fly vectors. Also, higher incidence of the disease in men could be due to factors such as their business and travel to endemic regions (Eid et al., 2018; Araujo et al., 2016; Khosravi et al., 2013).

The age distribution of the patients is presented in Table 1. The average of patients' age was 14.3 ± 16.7 years, ranging from 1 to 65 years. The disease is more prevalent in young age groups and patients under 10 years of age had the highest infection rate (64.3%), and the lowest infection rate (4.3%) was observed in people over 60 years old (Table 1). This finding is in agreement with the results of previous studies carried out in known ZCL endemic regions of Iran including Ilam, Kerman, Fars, and Golestan Provinces (Rezaee et al., 2020; Fakoorziba et al., 2011; Jorjani et al., 2019). Similarly, in studies conducted in Pakistan and Afghanistan the CL was more prevalent in young age groups (Brooker et al., 2004; Faulde et al., 2008). This may be because individuals of young age-group are more likely to be exposed to the sources of CL infection than others in areas with abundant reservoirs and vectors. In addition, the high prevalence of CL in young children may be due to the fact that these children are non-immune subjects who have been exposed for the first time in their life to the infective bite of the sand fly. Generally, the prevalence of leishmaniasis in the endemic areas decreases in those older than 15 years old, probably as a result of the acquired immunity (Al-Tawfiq and AbuKhamsin, 2004).

**Table 1:** The demographic characteristics of cutaneous leishmaniasis cases in the Hajiabad County, southeast of Iran.

Characteristics	Frequency	%
Gender		
Male	38	54.3
Female	32	45.7
Age group (years)		
≤10	45	64.3
11–20	6	8.6
21–30	6	8.6
31–40	5	7.1
51–60	5	7.1
>60	3	4.3
Residence place		
Urban	7	14.3
Rural	63	85.7

The majority of CL cases (85.7%) were reported from rural areas while only 14.3% of patients lived in urban areas (Table 1). The incidence of the disease in rural areas was six-times more than that of urban areas and a significant association was observed between the incidence of the disease and residential places of the patients (*p* = 0.0001). This finding is parallel with other studies in Iran which indicated high incidence of ZCL in rural areas (Norouzinezhad et al., 2016; Rostami et al., 2013; Mohammadi and Soltani, 2019; Nilforoushzadeh et al., 2014; Razmjou et al., 2009). ZCL has been common in different rural areas of Iran such as the northern parts of Isfahan Province, the northeastern plains near the Russian border, and the center of the country. However, the disease has recently spread to the south and southeast of Iran (Norouzinezhad et al., 2016). The prevalence of leishmaniasis in the rural areas of Hajiabad County indicates the endemicity of the disease in the County. In this regard, development of agricultural project, dumping of garbage and construction wastes around the residential areas, and houses plastered with clay and straw result in the attraction of sand flies and rodents near the villages (Chelbi et al., 2020). Furthermore, existence of animal shelters close to the living homes and construction of houses on farms near the colonies of rodents may increase the human contact with the sand flies and rodents which provides an efficient cycle for the transmission of the disease (Motazedian et al., 2006).

Clinical observation revealed that the majority of lesions were single (51.4%). Double lesions were observed in 22.9% of patients, and 25.7% of cases had multiple lesions. The highest number of lesions seen was 6. The average number of lesions was 2 per person. No significant difference was observed between the males and females in terms of number of skin lesions (*p* = 0.937) (Table 2).

**Table 2:** The clinical features of cutaneous leishmaniasis in the Hajiabad County, southeast of Iran.

Parameter	Male		Female		Total		*p*-value
	n	%	n	%	n	%	
Number of lesions							
1	20	52.6	16	50.0	36	51.4	0.937
2	9	23.7	7	21.9	16	22.9	
3	4	10.5	3	9.4	7	10.0	
≥ 4	5	13.2	6	18.8	11	15.7	
Total	39	100.0	32	100.0	70	100	

Location of the lesion							
Hands	18	47.4	16	50.0	34	48.7	0.984
Face	14	36.8	12	37.5	26	37.1	
Feet	3	7.9	2	6.25	5	7.1	
Other	3	7.9	2	6.25	5	7.1	
Total	38	100.0	32	100.0	70	100	

**Fig. 3:**
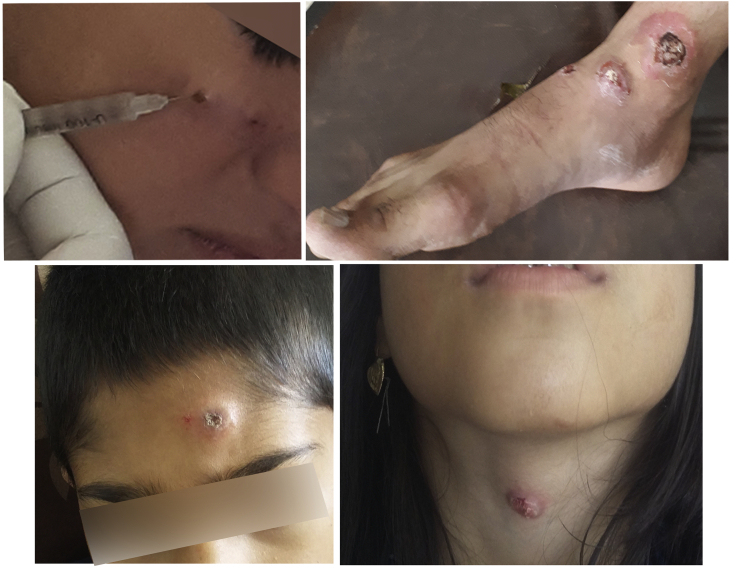
Cutaneous leishmaniasis lesions of patients in Hajiabad County, southeast of Iran.

As shown in Fig. 3, lesions were mainly found on the hands (48.7%), face (37.1%), and feet (7.1%) (Table. 2). There was no significant difference between males and females in terms of location of CL lesions (*p* = 0.984) (Table 2). The current finding is in accordance with the results of a previous study performed in the County which showed that the CL lesions were mainly on the exposed areas of the body, such as the face, hands, and feet (HanafiBojd et al., 2006). Such a distribution pattern of the lesions has been reported from different parts of Iran (Norouzinezhad et al., 2016; Razmjou et al., 2009; Karami et al., 2013). The distribution pattern may be due to short proboscis of sand flies, which makes them unable to bite through clothing; therefore, they attack exposed parts of the body including face, hands, and feet.

### 3.2 Sand fly fauna, species composition and abundance

During the study period, 832 adult sand fly specimens were collected and classified into ten *Sergentomyia* species and seven *Phlebotomus* species. Among the sand flies, 510(61.3%) were male and 322(38.7%) were female. *Phlebotomus* species included *P. papatasi*, *P. alexandri*, *P. salehi*, *P. caucasicus*, *P. mongolensis*, *P. sergenti*, and *P. bergeroti*. *Sergentomyia* species consisted of *S. sintoni*, *S. squamipleuris*, *S. baghdadis*, *S. clydei*, *S. hodgsoni*, *S. iranica*, *S. theodori*, *S. palestinensis*, *S. tiberiadis*, and *S. antennata* (Table 3). Among the collected *Phlebotomus* species, *P. papatasi* and *P. sergenti* are known as the primary vectors of CL and *P. alexandri*, *P. salehi*, and *P. caucasicus*, play the main role as the secondary vectors of CL in Iran (Yaghoobi-Ershadi, 2012; Shoraka et al., 2021). Furthermore, *P. alexandri* is known as secondary vector of visceral leishmaniasis (VL) in south of the country (Azizi et al., 2006).

**Table 3:** Species composition and relative abundance of phlebotomine sand flies collected from outdoors and indoors of Hajiabad County, southeast of Iran, 2019.

Species	Collection sites				Total	
	Indoors		Outdoors			
	n	%	n	%	n	%
*P. papatasi*	124	48.82	268	46.37	392	47.12
*P. alexandri*	14	5.51	56	9.69	70	8.41
*P. salehi*	13	5.11	39	6.75	52	6.25
*P. caucasicus*	6	2.36	18	3.11	24	2.88
*P. mongolensis*	6	2.36	16	2.77	22	2.64
*P. sergenti*	12	4.72	8	1.38	20	2.40
*P. bergeroti*	13	5.12	7	1.21	20	2.40
*S. sintoni*	12	4.72	38	6.57	50	6.01
*S. squamipleuris*	8	3.15	26	4.50	34	4.09
*S. baghdadis*	20	7.87	10	1.73	30	3.61
*S. clydei*	8	3.15	20	3.46	28	3.37
*S. hodgsoni*	0	0.00	28	4.84	28	3.37
*S. iranica*	8	3.15	10	1.73	18	2.16
*S. theodori*	4	1.57	10	1.73	14	1.68
*S. palestinensis*	2	0.79	12	2.08	14	1.68
*S. tiberiadis*	4	1.57	8	1.38	12	1.44
*S. antennata*	0	0.00	4	0.69	4	0.48
Total	254	100.00	578	100.00	832	100.00

Moreover, five species including *P. caucasicus*, *P. salehi*, *S. hodgsoni*, *S. squamipleuris*, and *S. iranica* were recorded for the first time in the Hajiabad County.

The sand fly, *P. papatasi* was the most abundant species, 392 specimens (47.12%) and found in almost all the localities studied, followed by *P. alexandri* (8.41%), *P. salehi* (6.25%), *S. sintoni* (6.01%), and *S. squamipleuris* (4.09%). The remaining 12 species accounted for 28.1% of the collected phlebotomine specimens (Table 3). In addition, *P. papatasi* was more prevalent in indoor (48.82%) and outdoor (46.37%) places. The other most prevalent species in indoor places were *S. baghdadis* (7.87%), *P. alexandri* (5.51%), and *P. bergeroti* (5.12), whereas *P. alexandri* (9.69%), *P. salehi* (6.75%), and *S. sintoni* (6.57%) were most common species in outdoor places (Table 3).

In this study, a total of 518 sand fly specimen including 17 species were collected from plain areas. *P. papatasi* was widely distributed and predominant species and accounted for 52.9% of all collected phlebotomine sand flies in the plain areas. The other three common species in plain area were *P. alexandri* (6.95%), *S. sintoni* (6.95%), and *P. salehi* (5.79%) (Table 4).

**Table 4:** Abundance of the sand flies in different topographical areas of Hajiabad County, southeast of Iran, 2019.

Species	Topographic areas				Total	
	plain		foothill			
	n	%	n	%	n	%
*P. papatasi*	274	52.90	118	37.58	392	47.12
*P. alexandri*	36	6.95	34	10.83	70	8.41
*P. salehi*	30	5.79	22	7.01	52	6.25
*P. caucasicus*	14	2.70	10	3.18	24	2.88
*P. mongolensis*	16	3.09	6	1.91	22	2.64
*P. sergenti*	6	1.16	14	4.46	20	2.40
*P. bergeroti*	10	1.93	10	3.18	20	2.40
*S. sintoni*	36	6.95	14	4.46	50	6.01
*S. squamipleuris*	18	3.47	16	5.10	34	4.09
*S. baghdadis*	10	1.93	20	6.37	30	3.61
*S. clydei*	10	1.93	18	5.73	28	3.37
*S. hodgsoni*	10	1.93	18	5.73	28	3.37
*S. iranica*	18	3.47	0	0.00	18	2.16
*S. theodori*	6	1.16	8	2.55	14	1.68
*S. palestinensis*	14	2.70	0	0.00	14	1.68
*S. tiberiadis*	6	1.16	6	1.91	12	1.44
*S. anntenata*	4	0.77	0	0.00	4	0.48
Total	518	100.00	314	100.00	832	100.00

According to the results, fourteen sand fly species were collected in foothill areas and the more prevalent species were *P. papatasi* (37.58%), followed by *P. alexandri* (10.83%), *P. salehi* (7.01%), and *S. baghdadis* (6.37%) (Table 4). During this study, three vectors of ZCL including *P. papatasi, P. salehi*, and *P. caucasicus* were more prevalent in the plain areas (Table 4).

In this study *S. iranica*, *S. palestinensis*, and *S. antennata* were collected in small numbers only in plain regions. In this regard, the number of sand flies collected was higher in the plain areas (*n* = 518) than in the foothill areas (Table 4).

The current study showed that *P. papatasi* was the most abundant species and mainly collected from plain areas. A previous study also indicated high prevalence of *P. papatasi* in the Hajiabad County (HanafiBojd et al., 2006). The sand fly has a wide distribution and reported from almost all Provinces of Iran (Karimi et al., 2014; Yaghoobi-Ershadi, 2012). In addition, *P. papatasi* is frequently distributed in arid and semiarid regions of the Old world from Morocco to Indian subcontinent (Hanafi-Bojd et al., 2018). In Iran, natural promastigote infections of *P.papatasi* have been found in almost all of ZCL foci and it is the proven vector of L. *major* in human and gerbils (Karimi et al., 2014; Yaghoobi-Ershadi, 2012; Parvizi et al., 2012).

In this study, *P. alexandri* was a common species which collected from all the topographic regions and represented 8.4% of all collected sand flies in the area tested. The sand fly, *P. alexandri* has been reported as a probable vector of zoonotic visceral leishmaniasis (ZVL) the south and southeast of the Iran (Azizi et al., 2006). This species is mostly distributed in mountainous areas and has been collected from almost all regions of Iran including highlands and plain areas (Yaghoobi-Ershadi, 2012; Kasap et al., 2019). This sand fly prefers warm areas with high relative humidity (Al-Jawabreh et al., 2004). Therefore, abundance of *P. alexandri* in the study area may be due to the favorable climatic conditions. *P. alexandri* has a wide distribution from Spain across to China and down to southern Ethiopia (Kasap et al., 2019).

In this study, *P. salehi* was mainly collected from outdoors in plain areas. The species was recorded for the first time in Hajiabad County. *P. salehi* had been previously reported from ZCL foci in the south and southeast of Iran (Azizi et al., 2012). The main distribution area of *P. salehi* is south and southeast of Iran, south of Pakistan, and northwest of India (Azizi et al., 2012; Davami et al., 2011). Sand fly, *P. salehi* is reported as a vector among gerbils in the enzootic cycle of L. *major* and it has been collected in rodent burrows in Baluchistan and Hormozgan Provinces in southeastern Iran (Yaghoobi-Ershadi, 2012; Azizi et al., 2012).

In the present study, *P. sergenti* was collected with low abundance and its frequency in the foothill areas was more than plain areas. The low abundance in the present study is comparable with previous study conducted in this County (HanafiBojd et al., 2006). Similar abundance has been reported for the species from Bandar Abbas, the neighboring County to Hajiabad (Mesghali, 1965). The low prevalence of *P. sergenti* might be attributed to the geography and environmental conditions of Hajiabad County. This species was mainly collected from mountainous areas and has a wide distribution in Iran and it has been reported from 26 provinces with three morphotypes (Yaghoobi-Ershadi, 2012; Shoraka et al., 2021; Moin-Vaziri et al., 2007). *P. sergenti* is known as the main vector of ACL in Iran and natural promastigote infections have been found in this species in two ACL foci including Mashhad and Isfahan cities (Yaghoobi-Ershadi, 2012; Shoraka et al., 2021).

The study findings showed that *P. caucasicus* was distributed with low frequency in both foothills and plain regions in the study area. This sand fly was also recorded for the first time in the Hajiabad County. The females of *P. caucasicus* cannot be separated morphologically from *P. mongolensis*, and *P. andrejevi*, and these are usually called *P. caucasicus* group (Yaghoobi-Ershadi, 2012; Shoraka et al., 2021). This phlebotomus preserves the enzootic cycle of L. *major* in gerbils as a secondary vector, and it is also known as a secondary vector of L. *major* to humans (Yaghoobi-Ershadi, 2012; Parvizi et al., 2012; Shoraka et al., 2021). The sand fly, *P. caucasicus* has been reported from different parts of Iran (Shoraka et al., 2021; Sofizadeh et al., 2018b). This sand fly is also widely distributed in Afghanistan, China, and the Russia (Shoraka et al., 2021).

In the present study seventeen species of sand flies were recorded in Hajiabad County, which shows the relatively high number of sand fly species in this County. It may be explained by development of agricultural activities and existence of animal shelters and rodent barrows close to human habitats that may provide breeding places, food sources, and ecological niches for sand fly species. Moreover, climatic conditions may have had influence on the proliferation and distribution of sand fly species in the study area.

## 4 Conclusion

Results of the present study indicated that CL is endemic in Hajiabad County and annual incidence rate of disease in the County is higher than the average incidence of the disease in Iran. Moreover, this study has revealed that there are five vectors of CL in the County. These findings can be used as a basis for implementation of interventions toward vector control, which may help in suppression of vector density, and consequently, control of CL in the study area. Moreover, appropriate educational programs focused on the transmission and preventive methods of the disease together with active surveillance to quickly detect and treat the cases are necessary to reduce the prevalence of CL in the area tested.

## Financial support

This study received financial support from Research and Technology Deputy of 10.13039/501100011917Hormozgan University of Medical Sciences (Project No.24/0892).

## Ethics statement

The patients were informed about the procedures and objectives of the study. Before the admission, an informed consent was taken from adult subjects and parents of children less than 15 years old. In addition, the subjects were informed that their participation is voluntary and they can withdraw from the study at any time. This study was confirmed by Ethical Committee of Hormozgan University Medical Sciences (Code No: IR.HUMS.REC.1398.251).

## Declaration of Competing Interest

The authors declare that they have no competing interests that could influence the work reported.
